# Real Aperture Radar Super-Resolution Imaging for Sea Surface Monitoring Based on a Hybrid Model

**DOI:** 10.3390/s23239609

**Published:** 2023-12-04

**Authors:** Ke Tan, Shengqi Zhou, Xingyu Lu, Jianchao Yang, Weimin Su, Hong Gu

**Affiliations:** School of Electronic and Optical Engineering, Nanjing University of Science and Technology, Nanjing 210094, China; zhoushengqi@njust.edu.cn (S.Z.); nust_luxyhenry@163.com (X.L.); yangjianchao999@126.com (J.Y.); suweimin@njust.edu.cn (W.S.); guhong666@126.com (H.G.)

**Keywords:** real aperture scanning radar, super-resolution imaging, sea clutter, Markov random field

## Abstract

In recent years, super-resolution imaging techniques have been intensely introduced to enhance the azimuth resolution of real aperture scanning radar (RASR). However, there is a paucity of research on the subject of sea surface imaging with small incident angles for complex scenarios. This research endeavors to explore super-resolution imaging for sea surface monitoring, with a specific emphasis on grounded or shipborne platforms. To tackle the inescapable interference of sea clutter, it was segregated from the imaging objects and was modeled alongside I/Q channel noise within the maximum likelihood framework, thus mitigating clutter’s impact. Simultaneously, for characterizing the non-stationary regions of the monitoring scene, we harnessed the Markov random field (MRF) model for its two-dimensional (2D) spatial representational capacity, augmented by a quadratic term to bolster outlier resilience. Subsequently, the maximum a posteriori (MAP) criterion was employed to unite the ML function with the statistical model regarding imaging scene. This hybrid model forms the core of our super-resolution methodology. Finally, a fast iterative threshold shrinkage method was applied to solve this objective function, yielding stable estimates of the monitored scene. Through the validation of simulation and real data experiments, the superiority of the proposed approach in recovering the monitoring scenes and clutter suppression has been verified.

## 1. Introduction

Real-aperture scanning radar (RASR) imaging technology is widely harnessed for scene monitoring and situational awareness, given its efficacy in rapid imaging and minimal configuration demands [1,2,3]. It attains a high range resolution by emitting linear frequency modulation (LFM) signals and subsequently employing pulse compression in the range direction. However, the angular resolution of RASR remains relatively low due to the spatial constraints on the aperture [4]. This limitation significantly restricts the potential application of RASR imaging. Fortunately, the azimuthal echo of RASR can be effectively conceptualized as the result of convolving the antenna pattern with the target’s scattering coefficients. Consequently, deconvolution technology can be used to facilitate estimation of the target’s scattering coefficient [5]. This approach is often referred to as a super-resolution technique. In recent years, researchers have increasingly employed this technology to enhance the resolution of RASR [6,7,8,9,10,11].

Presently, the published super-resolution imaging methods can be broadly classified into two distinct categories. Those in one category operate without specific prior information. Within this category, two notable methods are the iterative adaptive (IAA) approach [12,13] and the truncated singular value decomposition (TSVD) approach [14,15]. They are primarily used for global super-resolution estimation. The IAA method represents a non-parametric spectral estimation technique that excels at suppressing sidelobes while undertaking super-resolution estimation. However, it demands a substantial number of snapshots to achieve a high super-resolution ratio, making it computationally complex. On the other hand, the TSVD method is efficient in mitigating the impact of noise by discarding the singular value part of noise in the signal space, which is a convenient and fast approach to implement. However, its effectiveness in improving resolution is somewhat limited.

The super-resolution methods of the second category rely upon specific prior information to guide the deconvolution estimation. One commonly used approach in this category is the regularization method, which incorporates norm-based prior information based on linear observations. For instance, the classical Tikhonov method, which capitalizes on prior knowledge concerning the continuity of the imaging scene and penalizes target characteristics using the L2 norm, is capable of yielding relatively stable and continuous imaging results [16,17]. Nevertheless, the super-resolution ratio attained by this method tends to be relatively low. Additionally, methods incorporating the L1 norm and total variation (TV) norm have been explored. These norms can effectively distinguish sparse targets or preserve target contour information [18,19,20,21]. Nonetheless, the regularization methods may not offer a precise representation of sea clutter in the imaging process, rendering them susceptible to clutter interference.

Methods grounded in statistical optimization exhibit the capacity to adaptively incorporate prior information related to noise, clutter, and targets [22,23,24]. In reference [25], sparse prior knowledge was introduced to enhance the resolution of the super-resolution method. The measured data demonstrated their ability to distinguish critical targets. Reference [26] explores the use of mixed prior knowledge to reinforce the noise resilience of super-resolution methods. Additionally, the incorporation of the variational Bayesian method has been utilized in addressing outlier issues [27]. However, these methodologies are primarily tailored for ground imaging.

Pioneering works by researchers for sea surface targets were exhibited in references [28,29]. In their innovative approaches, sea clutter is characterized as Rayleigh distribution, while targets are assumed as Laplace and Lognormal distributions, respectively. These methodologies have shown promise in mitigating sea clutter while enhancing target resolution. Furthermore, reference [30] delves into sea clutter distribution at small incident angles, corroborating its feasibility through measured data. Unfortunately, for all these methods, no attention is given to the noise distribution, thus discounting the effect on the results. Reference [31] embraces both clutter and noise within the purview of the imaging process. Nevertheless, it resorts to a Gaussian mixture model for compounded noise and sea clutter, which leads to the deficiency of elegant physical interpretability. Notably, that study primarily conducted semi-physical experiments and lacks the robust validation necessary to assert its efficacy in real-world scenarios.

This manuscript presents an innovative super-resolution imaging method designed for sea surface scene monitoring on ground-based or shipborne platforms. It introduces a hybrid observation model that encompasses clutter and noise in addition to targeting distributions within a Bayesian criterion. Firstly, noise characterization is achieved through I/Q channels due to the orthogonal demodulation of the radar imaging system. Next, the sea clutter is represented as a Weibull distribution, providing the suitability for small incident angles and a reasonable dynamic range. Then, a joint modeling strategy is advocated by incorporating clutter distribution alongside I/Q channel noise within the maximum likelihood (ML) framework to eliminate the impact of clutter and noise. Additionally, to enhance the prior representation for target distribution, a Markov random field with two-dimensional representation capabilities is employed. This facilitates effective representation of the scene contours and the targets of interest simultaneously. Additionally, quadratic functions are utilized to stabilize the targets against outlier interference. The maximum a posterior criterion plays a central role, harmonizing the likelihood function and the prior distribution in formulating the final objective function. To address the complexity of this objective function, a robust fast iterative threshold shrinkage method is employed for meticulous computation.

The structure of this article is organized as follows: in Section 2, we establish the super-resolution imaging model for RASR. Subsequently, an objective function is derived based on a hybrid model in a Bayesian framework. Then, a fast iterative threshold shrinkage method is adopted to solve this objective function. Section 4 is dedicated to presenting the outcomes of numerical simulations and real data experiments. Finally, the conclusion is provided in Section 5.

## 2. Establishment of Echo Model for RASR

**Figure 1:**
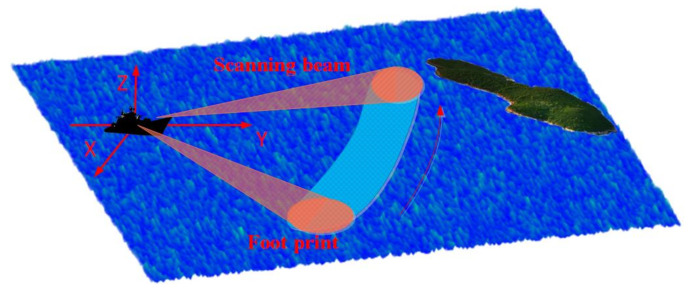
The imaging sketch of RASR.

**Figure 2:**
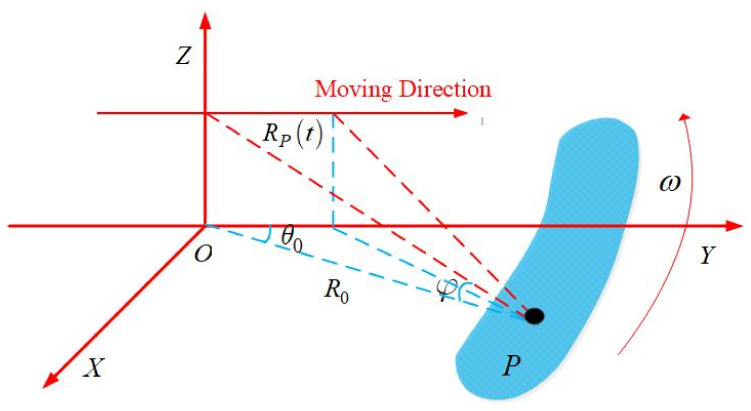
Geometric relationship diagram of RASR for a moving platform.

In this section, we initially formulate the echo model for the RASR. Figure 1 illustrates the operational configuration of a shipborne scanning radar in which the antenna revolves around the ship’s position and emits LFM signals for the purpose of sea surface detection. We assume that the transmitted LFM signal is

(1) $$s_{out}\left(\tau \right)=rect\left(\frac{\tau }{T_{r}}\right)\cos\left\{2\pi f_{c}\tau +\pi K\tau^{2}\right\}$$

where τ is the fast time variable, $T_{r}$ denotes the width of the pulse signal, $f_{c}$ is the carrier frequency, and *K* denotes the frequency modulation rate of LFM. $rect\left(\cdot \right)$ is a rectangle window function with width $T_{r}$. For target P at distance $R_{0}$, the echo signal within the scanning time can be written as

(2) $$s_{P0}\left(\tau ,t\right)=x_{0}h\left(t-t_{0}\right)\cos\left\{2\pi f_{c}\left(\tau -2R_{0}/c\right)+\pi Kr{\left(\tau -2R_{0}/c\right)}^{2}\right\}$$

where *t* is the slow time variable, $x_{0}$ denotes the scattering coefficient of the target, $h\left(t\right)$ is the radiation pattern of the antenna, $t_{0}$ is the center time while scanning target *P*, $f_{c}$ denotes the carrier frequency, and *c* is the light speed. After I/Q orthogonal demodulation, the baseband complex signal can be obtained as

(3) $$s_{P}\left(\tau ,t\right)=x_{0}h\left(t-t_{0}\right)\exp\left\{j\pi Kr{\left(\tau -2R_{0}/c\right)}^{2}\right\}\exp\left\{-j\frac{4\pi }{\lambda }R_{0}\right\}$$

where λ denotes the wavelength. Equation (3) represents the echo when the platform is stationary. While the platform is in motion, the distance $R_{0}$ varies with time. Referring to Figure 2, in the scenario where the platform moves uniformly along the y-direction during the imaging procedure, the distance history can be expressed as follows:

(4) $$R_{P}\left(t\right)=\sqrt{R_{0}^{2}+{\left(vt\right)}^{2}-2R_{0}vt\cos\theta_{0}\cos\varphi }$$

where *v* is the moving speed, $\theta_{0}$ denotes the initial azimuth angle of target *P*, and φ is the grazing angle. Due to the rapid scanning motion and relatively slow platform movement, it is reasonable to approximate the distance history as exhibiting linear variation [32]

(5) $$R_{P}\left(t\right)\approx R_{0}-vt\cos\varphi .$$

According to Equation (5), the echo formula can be rewritten as

(6) $$s_{P}\left(\tau ,t\right)=x_{0}h\left(t-t_{0}\right)\exp\left\{j\pi Kr{\left(\tau -2\left(R_{0}-vt\cos\varphi \right)/c\right)}^{2}\right\}\exp\left\{-j\frac{4\pi }{\lambda }\left(R_{0}-vt\cos\varphi \right)\right\}.$$

To facilitate spatial modeling and analysis, convert time variables into a spatial variable with t=θ/ω and τ=2r/c, where θ is the azimuth angle variable and *r* is the range variable. After conducting pulse compression and motion compensation, the echo expression for one target can be simplified as

(7) $$s_{P}\left(r,\theta \right)=x_{0}h\left(\theta -\theta_{0}\right)\mathrm{sinc}\left[\frac{2B}{c}\left(r-R_{0}\right)\right]\exp\left\{-j\frac{4\pi }{\lambda }\left(R_{0}-v\theta /\omega \cos\varphi \right)\right\}$$

For area targets with scattering coefficient $x(\bar{r},\bar{\theta })$, the echo can be written as the convolution of the scattering coefficient and the point target echo.

(8) $$s_{\Omega }\left(r,\theta \right)=\;\int \int \;\tilde{x}\left(\bar{r},\bar{\theta }\right)h\left(\theta -\bar{\theta }\right)\mathrm{sinc}\left\{\frac{2B}{c}\left(r-\bar{r}\right)\right\}d\bar{r}d\bar{\theta }\exp\left(j\varphi \left(\theta \right)\right)$$

where $\tilde{x}\left(\bar{r},\bar{\theta }\right)=x\left(\bar{r},\bar{\theta }\right)\exp\left\{-j\frac{4\pi }{\lambda }\bar{r}\right\}$ is the phased scattering coefficient, and $\varphi \left(\theta \right)=\frac{4\pi {\;}_{0}\;}{\lambda }v\theta /\omega \cos\varphi$ is a Doppler phase term, which can be eliminated through phase compensation. If we focus on the azimuth echo of a fixed distance $\bar{r}$, it can be found that the azimuth echo derives from the convolution of the target scattering coefficient and the azimuth antenna pattern

(9) $$s_{A}\left(\theta \right)=\int x\left(\bar{\theta }\right)h\left(\theta -\bar{\theta }\right)d\bar{\theta }$$

To further facilitate the numerical analysis, the convolution model in (9) can be discretized by a matrix vector form:

(10) s=Hx

where $s={\left[s_{1},\ldots ,s_{N}\right]}^{T}$ is the measured data, *N* denotes the length of the vector, $x={\left[x_{1},\ldots ,x_{N}\right]}^{T}$ is the discretized targets distribution, and H is the convolution matrix derived from the antenna pattern

(11) $$H=\left[h\left(\theta_{1}\right),h\left(\theta_{2}\right),\ldots ,h\left(\theta_{N}\right)\right]=\;\;{\left[\begin{array}{ccccccc} h_{1} & & & & h_{L} & \cdots & h_{2}\\ h_{2} & h_{1} & & & & \ddots & h_{3}\\ \vdots & & \ddots & & & & \vdots \\ h_{L} & & & & & & \\ & \ddots & & & & & \\ & & \ddots & & & & \\ & & & h_{L} & \cdots & h_{2} & h_{1} \end{array}\right]}_{N\times N},$$

where $h_{1},\ldots ,h_{L}$ is the discretized values of the antenna pattern.

Based on the convolution model, deconvolution techniques can be employed to enhance azimuth resolution. However, deconvolution technology is inherently an ill-posed problem, being highly sensitive to noise and errors. Even minor interference can cause the deconvolution result to significantly deviate from the true solution. To address this challenge, it is essential to utilize prior information within the imaging process to constrain the solution space.

## 3. Methodology

In this paper, the MAP framework is employed for super-resolution estimation, offering the capacity to flexibly and comprehensively incorporate prior information throughout the imaging process, encompassing noise, clutter, and targets. The MAP method stems from the Bayesian criterion, operating on the principle of determining the target estimate that maximizes the posterior probability density function when observation data are acquired. Firstly, we give the Bayesian formula

(12) $$P\left(x\left|s\right.\right)=\frac{P\left(s\left|x\right.\right)P\left(x\right)}{P\left(s\right)}.$$

where $P\left(\left.x\right|s\right)$ is the posterior probability density function with observed data s, $P\left(\left.s\right|x\right)$ is the likelihood probability density function, and $P\left(x\right)$ and $P\left(s\right)$ are the prior probability density function with x and s, respectively. The principle of the Bayesian criterion is to construct $P\left(\left.x\right|s\right)$ through the distribution of the noise, clutter, and targets. Then, the super-resolution estimation of the targets can be obtained by maximizing the posterior probability density function. Since $P\left(\left.s\right|x\right)$ is independent of x, maximizing $P\left(\left.x\right|s\right)$ is equivalent to maximizing the following equation:

(13) $$\hat{x}=\underset{x}{\arg\max}P\left(s\left|x\right.\right)P\left(x\right)$$

Typically, logarithmic functions are employed to transform product operations into additive ones, simplifying the analysis process.

(14) $$\hat{x}=\underset{x}{\arg\max}\;\;\;\;lnP\left(s\left|x\right.\right)+\ln P\left(x\right)$$

Given the monotonic nature of logarithmic functions, this transformation does not alter the solution for the targets. Equation (14) reveals that the objective function comprises two components: the initial part being the likelihood function, and the subsequent part representing prior information regarding the targets. Let us examine these two components individually.

### 3.1. Likelihood Function Based on the New Observation Model

The echo model established in the preceding section represents an idealized scenario. In practice, the observation process encompasses non-ideal factors, notably noise and clutter. This is particularly significant in oceanic imaging, where sea clutter significantly influences imaging outcomes. Consequently, it is imperative to holistically account for the effects of noise and clutter in the imaging process. Hence, the observation model requires revision as follows:

(15) s=Hx+n+c

where $n={\left[n_{1},\ldots ,n_{N}\right]}^{T}$ and $c={\left[c_{1},\ldots ,c_{N}\right]}^{T}$ are the noise vector and clutter vector. From Equation (15), we can see that, due to the randomness of n and c, the observed data s are also a random variable whose probability distribution is jointly determined by n and c.

To obtain the probability distribution of s, let us first examine the noise model. Considering single channel noise, noise is thermal noise generated by electronic motion that follows a Gaussian distribution with a mean of zero $n∼\left(0,\sigma^{2}\right)$. However, the radar data used for imaging comprise complex signals obtained through I/Q orthogonal channel demodulation. Consequently, to establish the probability distribution of observation data, we must engage in joint modeling of the I/Q channel echoes. This process has been elaborated on in our prior work [26] and will not be reiterated here. Therefore, when only the impact of noise random distribution is considered, the probability distribution of the amplitude of **s** can be formulated as follows:

(16) $$f\left(s\left|x\right.,n\right)=\prod_{i=1}^{N}f\left(s_{i}\right)=\prod_{i=1}^{N}\frac{s_{i}}{{\sigma_{n}}^{2}}e^{-\frac{{\left(s_{i}\right)}^{2}+{\left({\left(\mathrm{Hx}\right)}_{i}\right)}^{2}}{2{\sigma_{n}}^{2}}}J_{0}\left(\frac{s_{i}{\left(\mathrm{Hx}\right)}_{i}}{{\sigma_{n}}^{2}}\right)$$

Here, ${\sigma_{n}}^{2}$ is the noise variance, and $J_{0}\left(\cdot \right)$ is a zero-order Bessel function. In fact, the random variable c would also affect the distribution of s. Therefore, it is also necessary to consider the distribution of sea clutter based on Equation (16). Even K distribution is more realistic among various sea clutter distributions [33], where the complexity of its distribution increased the difficulty in constructing the objective function, while the Weibull distribution has demonstrated superiority for describing the distribution of sea clutter at low grazing angles as well as the capacity to offer a reasonable dynamic range [34,35]. In addition, in the analysis of real data, we found that the measured sea clutter can fit well to a Weibull distribution by estimating appropriate model parameters. Therefore, we have opted for the Weibull distribution in this study to characterize the sea clutter distribution. The expression for the Weibull distribution is firstly stated as follows:

(17) $$f\left(c\right)=\prod_{i=1}^{N}f\left(c_{i}\right)=\prod_{i=1}^{N}\frac{\upsilon }{b^{\upsilon }}c_{i}^{\upsilon -1}e^{-\frac{c_{i}^{\upsilon }}{b^{\upsilon }}}\;\;\;\;\;\;\;\;\;\;\;\;\;\;\;c_{i}>0$$

where υ is the shape parameter, with a value range of (0,2). This mirrors the trailing characteristics of the Weibull distribution, where a smaller value of υ corresponds to a more pronounced trailing of the clutter distribution and *b* is the scale parameter. If only clutter is considered, the probability distribution of s can be formulated as follows:

(18) $$f\left(s\left|x\right.,c\right)=\prod_{i=1}^{N}f\left(c_{i}\right)=\prod_{i=1}^{N}\frac{\upsilon }{b^{\upsilon }}{\left(s_{i}-{\left(\mathrm{Hx}\right)}_{i}\right)}_{}^{\upsilon -1}e^{-\frac{{\left(s_{i}-{\left(\mathrm{Hx}\right)}_{i}\right)}_{}^{\upsilon }}{b^{\upsilon }}}\;\;\;\;\;\;\;\;\;\;\;\;\;\;\;c_{i}>0$$

In fact, the probability distribution of sea surface targets is determined by the internal noise of the system as well as the sea clutter. Therefore, taking into account the clutter distribution and the noise model, the condition probability function of the observed data, i.e., the likelihood function, has the following form:

(19) $$lnP\left(s\left|x,n,c\right.\right)=\;ln\prod_{i=1}^{N}\left(\frac{s_{i}}{{\sigma_{n}}^{2}}e^{-\frac{{\left(s_{i}\right)}^{2}+{\left({\left(\mathrm{Hx}\right)}_{i}\right)}^{2}}{2{\sigma_{n}}^{2}}}J_{0}\left(\frac{s_{i}{\left(\mathrm{Hx}\right)}_{i}}{{\sigma_{n}}^{2}}\right)\frac{\upsilon }{b^{\upsilon }}{\left(s_{i}-{\left(\mathrm{Hx}\right)}_{i}\right)}_{}^{\upsilon -1}e^{-\frac{{\left(s_{i}-{\left(\mathrm{Hx}\right)}_{i}\right)}_{}^{\upsilon }}{b^{\upsilon }}}\right)$$

### 3.2. Target Prior Information

**Figure 3:**
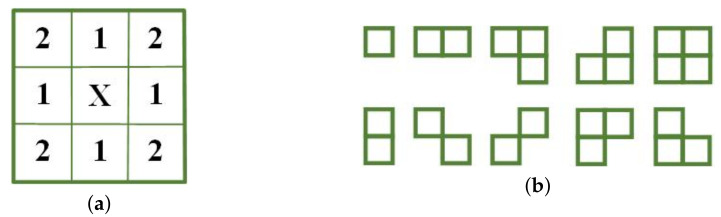
(**a**) The second-order neighborhood. (**b**) Clusters for the second-order neighborhood.

In the context of super-resolution imaging for surveillance scenes, the objective extends beyond merely distinguishing targets, and also encompasses the need to restore the scene’s finer details. Compared to the randomness of noise, the image’s texture exhibits a certain degree of correlation and regularity. To harness this characteristic and enhance super-resolution outcomes, we have chosen to employ the MRF model, which focuses on considering the conditional distribution of each pixel concerning its neighboring pixels. This model effectively captures the local statistical characteristics of the image, all without assuming that the image is stationary. Firstly, define a neighborhood system $\hbar^{o}\left(x\left(i,j\right)\right)$ on a discrete two-dimensional random field, where *o* is the neighborhood order. As shown in Figure 3a, each pixel with the same distance from pixel $x\left(i,j\right)$ belongs to the same neighborhood system. The first-order neighborhood system and the second-order neighborhood system of $x\left(i,j\right)$ is composed of the eight pixels and can be written as

(20) $$\hbar^{2}\left(x\left(i,j\right)\right)=\left[\begin{array}{ccc} x\left(i-1,j-1\right) & x\left(i-1,j\right) & x\left(i-1,j+1\right)\\ x\left(i,j-1\right) & & x\left(i,j+1\right)\\ x\left(i+1,j-1\right) & x\left(i+1,j-1\right) & x\left(i+1,j+1\right) \end{array}\right].$$

Pixels belonging to the same neighborhood system form different clusters based on their positions relative to $x\left(i,j\right)$. Figure 3b shows the types of clusters constructed from the permutation of the relative position of the second-order neighborhood system. A cluster represents a basic correlation between pixels or a basic composition of textures.

Furthermore, the random field defined on this neighborhood system can be acquired according to the Markov–Gibbs equivalence, which can be written as

(21) $$p\left(x\right)=\frac{1}{Z}e^{\left[-U\left(x\right)\right]}\;\;\;\;\;\;\;\;\;\;\;\;\;\;\;\;\;\;\;x\in \Omega$$

where $U\left(x\right)$ is called the energy function, Ω is the set of the clusters, and *Z* is a normalization parameter. The energy function commonly employed is the Gaussian function. Even though the Gaussian–Markov random field facilitates relatively straightforward analyses, the outcomes tend to be characterized by smoothness and a limited capacity to preserve image edges. Consequently, this study opts for an alternative approach, employing a non-Gaussian–Markov random field as the potential function [36]. This choice takes the following form:

(22) $$p\left(x\right)=\frac{1}{Z}e^{\left\{-\frac{1}{\tau }\sum_{c\in \Omega }\left|d_{c}^{}\left(x\right)\right|\right\}}$$

where τ is a temperature coefficient, and $d_{c}^{}\left(x_{i,j}\right)$ denotes a derivative operation, which is

(23) $$\begin{array}{c} {d_{i,j}}^{0}=x_{i,j+1}-2x_{i,j}+x_{i,j-1}\\ {d_{i,j}}^{1}=\frac{1}{2}\left(x_{i-1,j+1}-2x_{i,j}+x_{i+1,j-1}\right)\\ {d_{i,j}}^{2}=x_{i-1,j}-2x_{i,j}+x_{i+1,j}\\ {d_{i,j}}^{3}=x_{i-1,j-1}-2x_{i,j}+x_{i+,j+1} \end{array}$$

Furthermore, to ensure the stability of the solution throughout the solving process, a square penalty has been introduced. This measure can prevent the occurrence of outliers during the solution process. Therefore, the objective prior term can be written as

(24) $$\ln p\left(x\right)=\ln\prod_{i=1}^{N}\frac{1}{Z}e^{\left\{-\frac{1}{\tau }\sum_{c\in C}\left|d_{c}^{n}\left(x_{i}\right)\right|\right\}}\frac{1}{\sqrt{2\pi }\gamma }e^{\left(-\frac{{x_{i}}^{2}}{2\gamma^{2}}\right)}.$$

where γ is the standard deviation of the target distribution. Through substituting Equations (19) and (24) into Equation (14), the objective function of super-resolution imaging for RASR can be obtained:

(25) $$\begin{array}{c} J\left(x\right)=ln\prod_{i=1}^{N}\left(\frac{s_{i}}{{\sigma_{n}}^{2}}e^{-\frac{{\left(s_{i}\right)}^{2}+{\left({\left(\mathrm{Hx}\right)}_{i}\right)}^{2}}{2{\sigma_{n}}^{2}}}J_{0}\left(\frac{s_{i}{\left(\mathrm{Hx}\right)}_{i}}{{\sigma_{n}}^{2}}\right)\frac{\upsilon }{b^{\upsilon }}{\left(s_{i}-{\left(\mathrm{Hx}\right)}_{i}\right)}_{}^{\upsilon -1}e^{-\frac{{\left(s_{i}-{\left(\mathrm{Hx}\right)}_{i}\right)}_{}^{\upsilon }}{b^{\upsilon }}}\right)\\ +\ln\prod_{i=1}^{N}\frac{1}{Z}e^{\left\{-\frac{1}{\tau }\sum_{c\in C}\left|d_{c}^{n}\left(x_{i}\right)\right|\right\}}\frac{1}{\sqrt{2\pi }\gamma }e^{\left(-\frac{{x_{i}}^{2}}{2\gamma_{}^{2}}\right)} \end{array}$$

Expanding the logarithmic function, the objective function can be simplified as

(26) $$\begin{array}{c} \hat{x}=\underset{x}{\arg\max}\sum_{i=1}^{N}\ln J_{0}\left(\frac{s_{i}{\left(\mathrm{Hx}\right)}_{i}}{{\sigma_{n}}^{2}}\right)-\sum_{i=1}^{N}\frac{{\left({\left(\mathrm{Hx}\right)}_{i}\right)}^{2}}{2{\sigma_{n}}^{2}}+\left(\upsilon -1\right)\ln\left(s_{i}-{\left(\mathrm{Hx}\right)}_{i}\right)\\ -\frac{{\left(s_{i}-{\left(\mathrm{Hx}\right)}_{i}\right)}_{}^{\upsilon }}{b^{\upsilon }}-\eta_{1}\sum_{i=1}^{N}\sum_{n=1}^{4}\left(\left|d_{c}^{n}\left(x_{i}\right)\right|\right)-\eta_{2}\sum_{i=1}^{N}{x_{i}}^{2} \end{array}$$

where $\eta_{1}=\frac{1}{\tau }$ and $\eta_{2}=\frac{1}{2\gamma_{}^{2}}$ have similar roles to the parameters in the regularization method in being used to determine the weight values of these two prior information items. The L-curve method of regularizing parameters can be used to determine these two parameter values. Specifically, the $\eta_{1}$ can be firstly fixed by an experience value and then the value of $\eta_{2}$ can be determined through the L-curve method [37]. Then, fix $\eta_{2}$ by the obtained value and modify the value of $\eta_{1}$ through either in the L-curve method. Finally, the super-resolution estimation finds the targets x that maximize the objective function.

### 3.3. Solution to the Objective Function

Given that the objective function (26) is a complex nonlinear function, linear solutions are infeasible. To tackle this challenge, this article employs the iterative optimization strategy. Among various iterative methods, the fast ISTA method is chosen for its fast convergence performance and noise resistance stability. Firstly, the iterative process starts with the computation of the gradient of the objective function. Since the absolute value term in Equation (26) is nondifferential, it should be firstly approximated by a smooth term:

(27) $$\sum_{i=1}^{N}\sum_{n=1}^{4}\left(\left|d_{c}^{n}\left(x_{i}\right)\right|\right)=\sum_{i=1}^{N}\sum_{n=1}^{4}\left(\sqrt{\left({\left(d_{c}^{n}\left(x_{i}\right)\right)}^{2}+\varepsilon \right)}\right)$$

where ε≥0 is a minuscule value. Then, the gradient function can be calculated as

(28) $$\begin{array}{c} \nabla J\left(x\right)=\frac{1}{{\sigma_{n}}^{2}}H^{T}\left[\frac{J_{1}\left(\frac{s_{i}{\left(\mathrm{Hx}\right)}_{i}}{{\sigma_{n}}^{2}}\right)}{J_{0}\left(\frac{s_{i}{\left(\mathrm{Hx}\right)}_{i}}{{\sigma_{n}}^{2}}\right)}⊙s\right]-\frac{1}{{\sigma_{n}}^{2}}H^{T}\mathrm{Hx}+\left(\nu -1\right)\frac{H^{T}}{s-H^{T}x}-\;\;\frac{\nu }{b^{\upsilon }}H^{T}{\left(s-H^{T}x\right)}^{\nu -1}\\ -\eta_{1}\sum_{n=1}^{4}diag\left\{{\left({\left|{\left(d_{c}^{n}x\right)}_{m}\right|}^{2}+\varepsilon \right)}^{-\frac{1}{2}}\right\}d_{c}^{n}x-\eta_{2}x\; \end{array}$$

where ${\left(\cdot \right)}_{m}$ is the mth element of the vector in the bracket and $diag\left(\cdot \right)$ is a diagonal matrix. Then, the iterative direction used to search for the optimal solution is given by

(29) $$x^{k+1}={ℜ}_{\delta }\left\{x^{k}+\beta \nabla J\left(x^{k}\right)\right\}$$

where $x^{k}$ denotes the estimated targets, and β is the step size; to guarantee the convergence of the iteration, it is no greater than $2/∥H^{T}H∥$. ${ℜ}_{\delta }:R^{N}\to R^{N}$ is the shrinkage–thresholding operation

(30) $${ℜ}_{\delta }\left(x_{i}\right)=\left\{\begin{array}{c} 0\;\;\;\;\;\;\;\;\;\;\;\;\;\;\;\;\;\;\;\;\;\;\;\;\;\;\;\;\;\;\;\;\;\;\;\;\;\;\;\;\;\;\;\;\;\;\;\;\;\;\;\;\;\;\;\;\;\;\;\;\;\;\;\;\;\;,\;\;\;\;\;\;\left|\sigma_{i}\right|\leq \delta \\ x_{i}-\delta \mathrm{sgn}\left(x_{i}\right)\;\;\;\;\;\;\;\;,\;\;\;\;\;\;otherwise \end{array}\right.$$

It is worth noting that the shape parameter υ and scale parameter *b* should first be estimated using the following equation:

(31) $$\left\{\begin{array}{c} \;\frac{\Gamma \left(1+2/\hat{\upsilon }\right)}{\Gamma^{2}\left(1+1/\hat{\upsilon }\right)}\;\;\;\;\;=\;\;\;\frac{m_{2}^{2}}{m_{1}^{2}}\;\;\;\;\\ \hat{b}=\frac{m_{1}}{\Gamma \left(1+1/\hat{\upsilon }\right)} \end{array}\right.$$

According to Equation (31), the shape and scale parameters can be estimated from the sea clutter, where $\Gamma \left(\cdot \right)$ is the gamma function, and ${\hat{m}}_{1}$ and ${\hat{m}}_{2}$ are determined though ${\hat{m}}_{1}=\frac{1}{N}\sum_{i=1}^{N}\left|x_{i}\right|$, ${\hat{m}}_{2}=\frac{1}{N}\sum_{i=1}^{N}{x_{i}}^{2}$. In addition, the noise variance can be estimated from the signal without transmitting the signal.

Normally, achieving a high super-resolution ratio requires a large number of iterations. Therefore, a fast ISTA version is adopted [38]. Then the implementation steps of the proposed method for sea surface targets imaging are given in Algorithm 1. Firstly, assume that the real-beam two-dimensional echo is $S={\left[s_{1},\cdots ,s_{j},\cdots ,s_{M}\right]}^{T}$, where $s_{j}$ is the echo data of the j-th distance unit and M is the number of distance units to be processed. $X^{k}={\left[x_{1}^{k},\cdots ,x_{j}^{k},\cdots ,x_{M}^{k}\right]}^{T}$ is the k-th iteration result, and $x_{j}^{k}$ is the k-th iterative results of the j-th distance unit.

This proposed hybrid-model super-resolution method can address the challenges of sea surface scene monitoring, delivering effective and stable results in terms of resolving targets and restoration of scene details.
**Algorithm 1:** The implementation steps of the proposed hybrid-model method.**Initialization:** Estimate noise variance ${\sigma_{n}}^{2}$ and determine the regularization                    parameters $\eta_{1}$ and $\eta_{2}$                    Estimate shape $\hat{\upsilon }$, scale parameters of clutter $\hat{b}$**Step 1:** Initialize acceleration step size: $t_{1}=1$      Give S to the initial iterative matrix $X^{0}$ and prediction matrix $Y^{0}$**Step k ( k>1 ):**      **Repeat** j = 2 : M − 1      Extract the j-th distance unit data $s_{j}$ from S      Calculate the next iterative value $x_{j}^{k+1}$ using the predicted result $y_{j}^{k}$ by iteration formula      $x_{j}^{k+1}={ℜ}_{\delta }\left\{y_{j}^{k}+\beta \left(\begin{array}{c} \frac{1}{{\sigma_{n}}^{2}}H^{T}\left[\frac{J_{1}\left(\frac{s_{i,j}{\left({\mathrm{Hy}}_{j}^{k}\right)}_{i}}{{\sigma_{n}}^{2}}\right)}{J_{0}\left(\frac{s_{i,j}{\left({\mathrm{Hy}}_{j}^{k}\right)}_{i}}{{\sigma_{n}}^{2}}\right)}⊙s_{j}\right]-\frac{1}{{\sigma_{n}}^{2}}H^{T}{Hy}_{j}^{k}+\left(\nu -1\right)\frac{H^{T}}{s-H^{T}y_{j}^{k}}\\ -\;\;\frac{\nu }{b^{\upsilon }}H^{T}{\left(s_{j}-H^{T}y_{j}^{k}\right)}^{\nu -1}-\eta_{1}\sum_{n=1}^{4}diag\left\{{\left({\left|{\left(d_{c}^{n}y_{j}^{k}\right)}_{m}\right|}^{2}+\varepsilon \right)}^{-\frac{1}{2}}\right\}d_{c}^{n}y_{j}^{k}-\eta_{2}y_{j}^{k}\; \end{array}\right)\right\}$      **Until** (j = M − 1)      Update the acceleration step size      $t_{k+1}=\frac{1+\sqrt{1+4t_{k}^{2}}}{2}$      Update the prediction matrix $Y^{k}$      $Y^{k}=X^{k}+\left(\frac{t_{k}-1}{t_{k+1}}\right)\left(X^{k}-X^{k-1}\right)$**Until (convergence)****Export the final image**$X^{k}$

## 4. Numerical Results

In this section, both numerical simulation and measured data are used to verify the effectiveness of the proposed method.

### 4.1. Simulation Experiment

**Figure 4:**
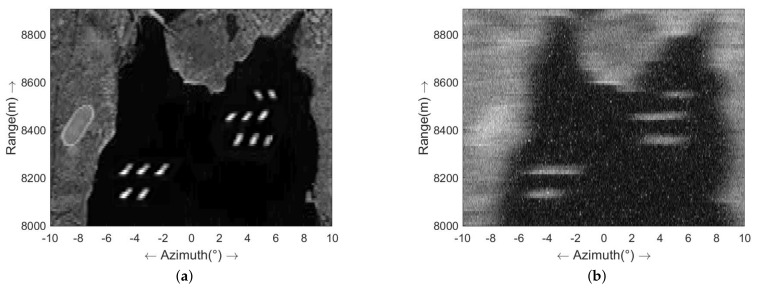
Simulation experiment (**a**) simulated scene; (**b**) real-beam echo.

Figure 4 illustrates the simulated scenario, portraying a harbor with several ships docked. In this scenario, we presume that a radar system is situated on a distant ship and emits LFM signals for harbor imaging. The simulation parameters are outlined in Table 1. Figure 4b showcases the real-beam image acquired after range pulse compression and motion compensation, featuring a signal-to-noise ratio (SNR) of 13 dB. To simulate the realistic scenario, sea clutter is introduced to the sea area. To simulate the sea wave, sea clutter is generated by a spherically invariant random process (SIRP) [39], with a Gamma-distributed process modulating the spectrum of the speckle. The Douglas sea state is set as 3 and the radar parameters used are displayed in Table 1. The final signal-to-clutter-noise ratio (SCNR) is equal to 10 dB. From Figure 4b, it can be observed that distinguishing the harbor’s contour from the ships is challenging due to the low azimuth resolution.

**Figure 5:**
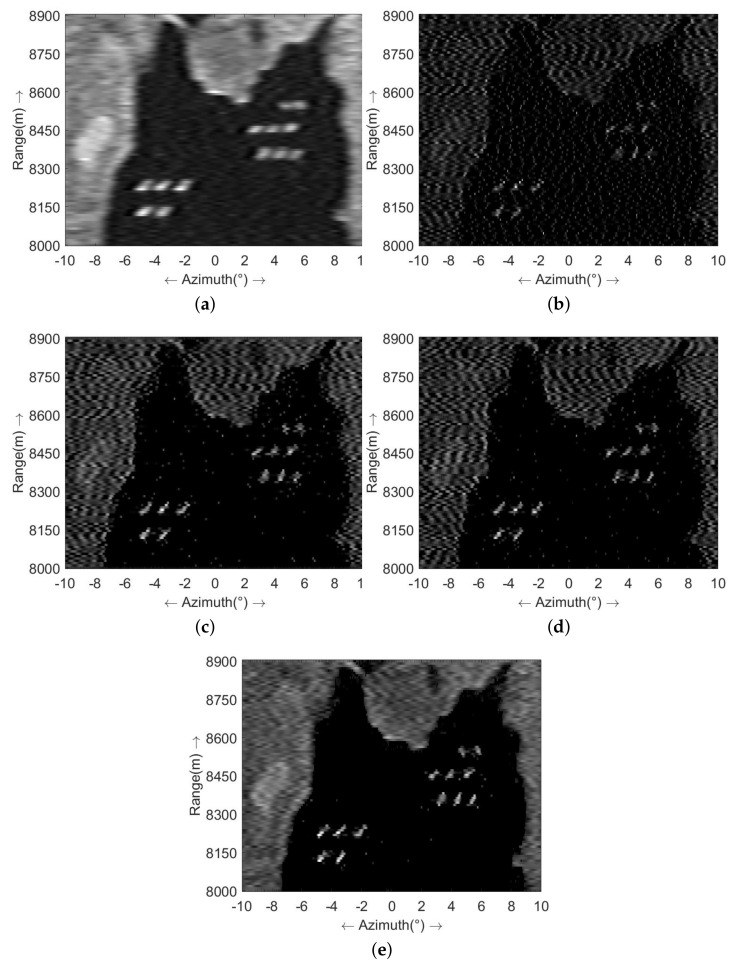
Comparison of super-resolution results in the case of SCNR=10 dB (**a**) Tikhonov method; (**b**) sparse-MAP method; (**c**) MRF-MAP method; (**d**) Weibull-MAP method; (**e**) the proposed hybrid-model method.

Subsequently, we applied the Tikhonov method, the sparse-MAP method, the MRF-MAP method introduced by our previous work, the sea surface super-resolution imaging method delineated in reference [30], which is named as Weibull-MAP, and the hybrid-model super-resolution method introduced in this study to process the echoes. The outcomes are sequentially shown in Figure 5. Figure 5a shows the results of the Tikhonov method. Notably, super-resolution processing has improved the azimuthal resolution and the scene’s contours are becoming clearer. However, due to its excessive smoothing effect, the method struggles with resolving ship targets that are closely located. Figure 5b displays the outcomes of the sparse-MAP method. The utilization of sparse priors has separated the ship thoroughly compared to the Tikhonov method. However, the presence of clutter and noise has given rise to numerous extraneous ripples after processing.

Figure 5c showcases the outcomes of the MRF-MAP method. This technique leverages MRF prior information, resulting in clearer target contour restoration and the elimination of substantial ripples within the scene. However, as clutter is not explicitly addressed, sea surface targets still undergo some deformation due to clutter influence. Figure 5d shows the result of Weibull-MAP. In this approach, the clutter is modeled as Weibull distribution, which leads to a clearer restoration of the sea surface targets compared to the MRF-MAP method. Nevertheless, some scattered interference remains present on the sea surface due to the absence of the noise model, and the capability to restore the scene contours is somewhat limited. Figure 5e provides the results of the hybrid-model method introduced in this paper. Evidently, owing to the integrated modeling of noise and clutter, the super-resolution results enable precise differentiation of sea targets, with optimal restoration of the harbor and playground contours.

**Figure 6:**
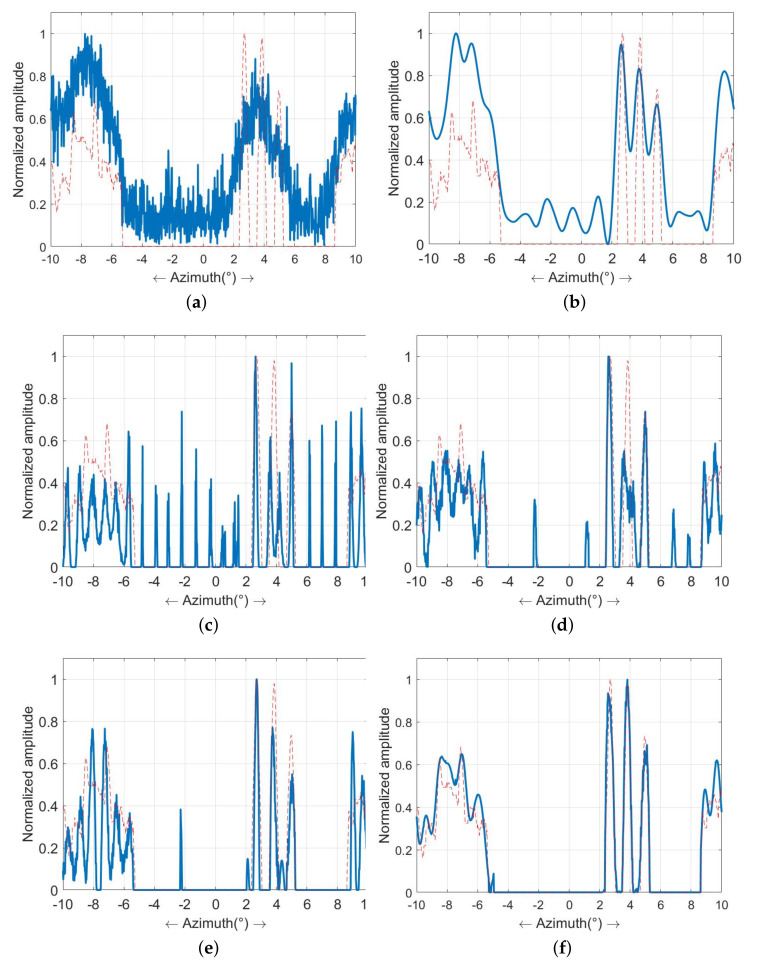
The profile of super-resolution outcomes in the case of SCNR=10 dB: (**a**) echo; (**b**) Tikhonov method; (**c**) sparse-MAP method; (**d**) MRF-MAP method; (**e**) Weibull-MAP method; (**f**) the proposed hybrid-model method.

Figure 6 illustrates the profile of the results presented in Figure 5 at a range of 8445 m. In these visualizations, the red line denotes the original target, while the blue line represents the super-resolution outcome. Analyzing the profile map, it is evident that the Tikhonov method exerts the most smoothing effect on the results at the cost of a relatively low resolution. The sparse-MAP method exhibits commendable resolution capability for targets but with the notable introduction of false targets, where the amplitudes of some false targets even surpass the targets. Both the MRF-MAP method and the Weibull-MAP effectively distinguish targets and mitigate the emergence of false targets to a certain extent. However, the method proposed in this study outperforms its counterparts in target recognition, false target suppression, and scene contour restoration.

To provide a more quantitative comparison of the results obtained from different super-resolution methods, relative error (ReErr) and structural similarity (SSIM) were used for evaluation. The definitions of ReErr and SSIM can be found in reference [23]. ReErr measures the energy difference between the super-resolution results and the real scene, where lower ReErr values indicate that the results are closer to the real scene. SSIM quantifies the structural similarity between the super-resolution results and the real scene. Higher SSIM values indicate greater similarity in the structure to the real scene. Additionally, when calculating these two metrics, more weight is given to the ship area compared to the background. This emphasizes the significance of key targets in the evaluation process. The numerical comparison results are presented in Table 2, where it is evident that the proposed method achieves the lowest ReErr and the highest SSIM, indicating its superiority in terms of both energy accuracy and structural similarity compared to the other methods. Moreover, the proposed method can possess a relatively low ReErr value and a high SSIM value even when the SCNR is low.

### 4.2. Real Data Experiment

**Figure 7:**
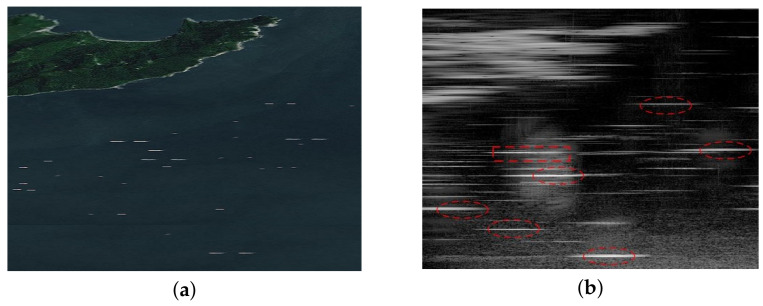
Real data experiment: (**a**) the imaging scene of the physical experiment; (**b**) real-beam echo.

In this section, we apply the proposed method to process the measured data to validate the effectiveness of the proposed hybrid-model method. Figure 7a illustrates a schematic diagram of the imaging scene, sourced from Google Maps. In this scene, a small island is situated above, and several ships are depicted as cruising on the sea surface, although this depiction is purely schematic drawing. A radar is installed on another small island and scans the sea surface for real-beam imaging. The radar works at 9.4 GHz and scans with a beam width of 2.5°. The transmitted LFM signal employed in this scenario possesses a bandwidth of 2.5 MHz. The sea surface wind and waves on the day of the experiment were relatively small and, upon investigation, the sea waves on that day were light waves ranging from 0.6 m to 0.9 m.

The real-beam image obtained is presented in Figure 7b. Due to its low azimuth resolution, the contour of the island in the upper left corner of the scene appears relatively coarse. The echoes of suspected ship targets in the echoes are marked with a rectangle box and are elliptic. Due to the low azimuth resolution, it is impossible to distinguish whether these echoes contain multiple ships.

**Figure 8:**
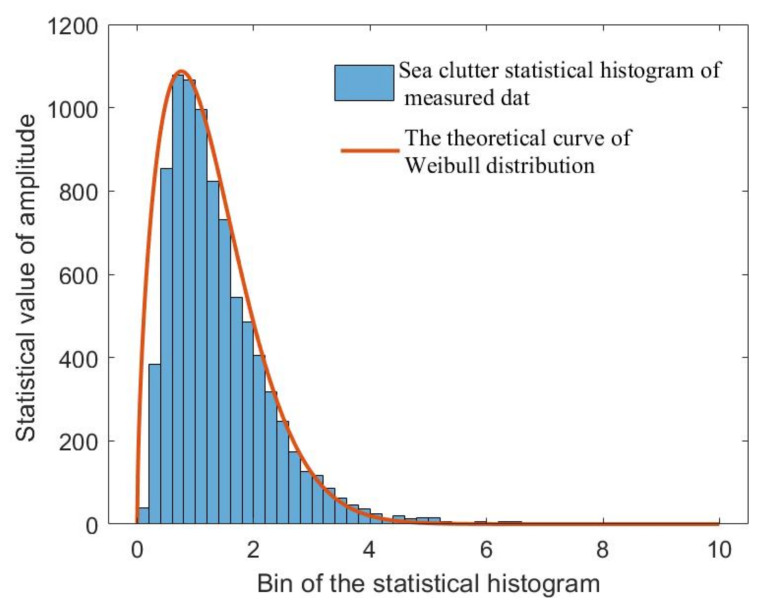
Distribution curve of the experimental sea clutter amplitude.

Then, the model parameters of sea clutter are evaluated through Equation (31), and the results are estimated to be υ=1.6 and b=1.4, respectively. Furthermore, amplitude statistics are conducted on the clutter in the sea surface area without targets. The statistical results are shown in Figure 8, in which the amplitude distribution of sea clutter is consistent with the Weibull distribution with the obtained model parameters.

**Figure 9:**
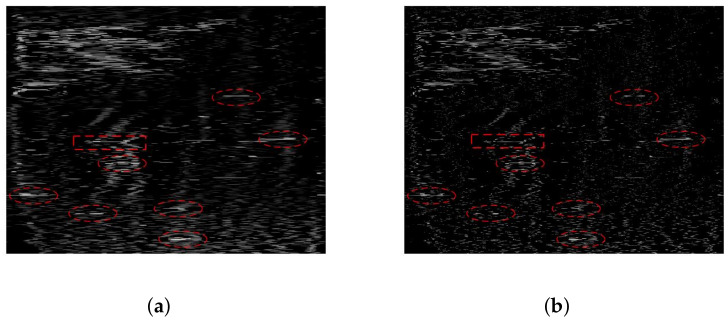

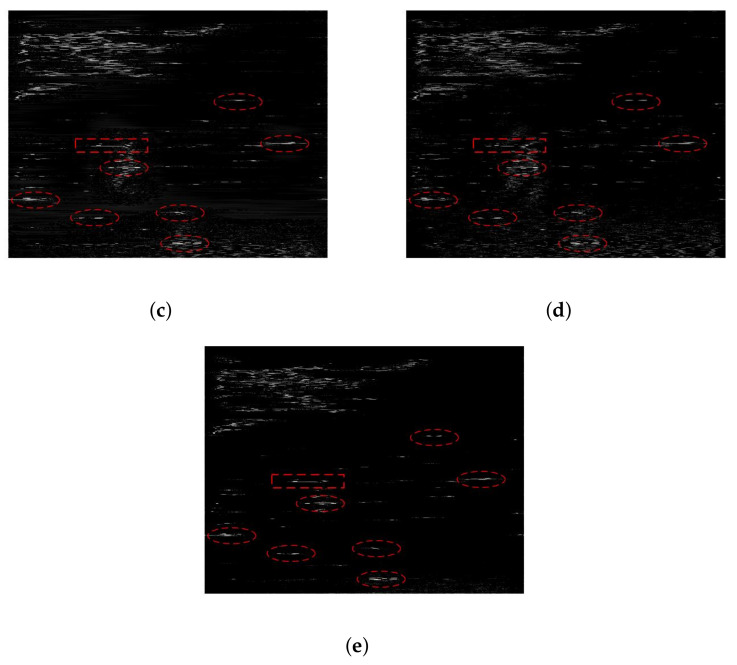
The super-resolution results of measured data: (**a**) Tikhonov method; (**b**) sparse-MAP method; (**c**) MRF-MAP method; (**d**) Weibull-MAP method; (**e**) the proposed hybrid-model method.

Next, we apply the method proposed in this paper along with four comparative methods conducted on the real-beam data. The results are displayed in Figure 9a–e. Figure 9a illustrates the results of the Tikhonov method, which demonstrates an improvement in resolution. However, there is a considerable amount of clutter and interference present in the scene. Figure 9b presents the results of the sparse MAP method, showcasing a notable enhancement in resolution. Nevertheless, the clutter in the result is scattered into isolated points, which significantly affects the final outcome. Figure 9c,d depict the results of the MRF-MAP and Weibull-MAP methods, respectively. In both cases, there is a substantial increase in resolution, and the background appears relatively clean. However, there is still a significant amount of clutter residue at the bottom of the scene. Figure 9e displays the results of the proposed method in this paper, showcasing its strong resolution capabilities in recovering the original scene. Not only is the outline of the island clearer, but the resolution of the marked targets has also been significantly improved. Some adjacent ships that cannot be distinguished in real-beam echo can be separated in Figure 9e. Moreover, the background of the entire scene remains relatively clean, indicating the method’s ability to distinguish in clutter suppression.

**Figure 10:**
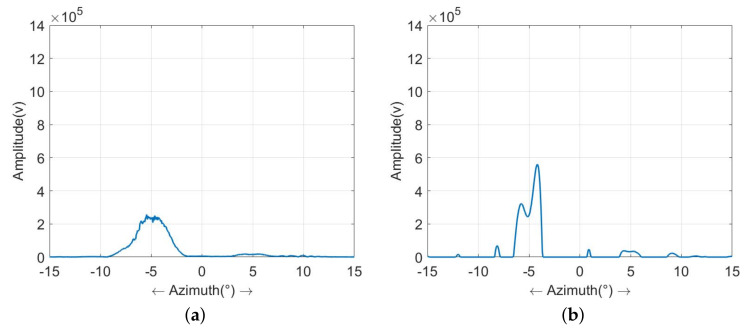

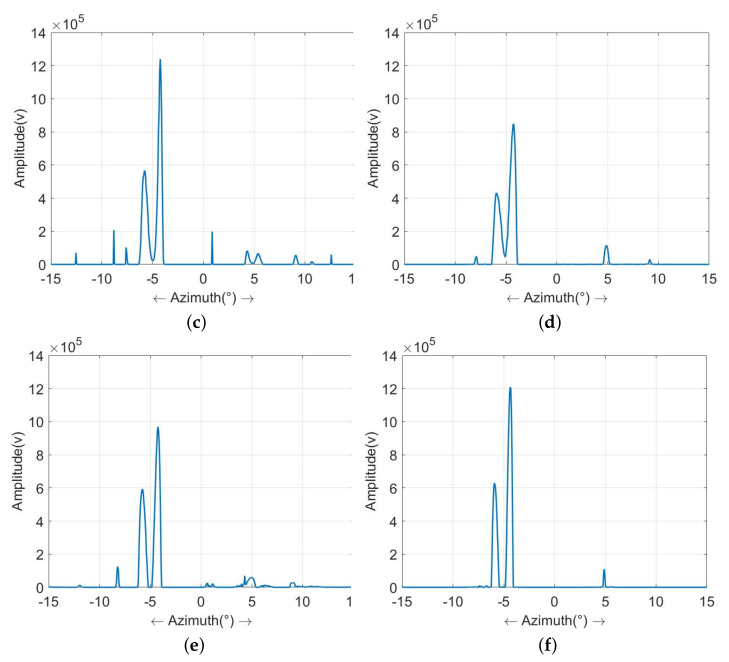
Profiles of the super-resolution results of measured data: (**a**) echo; (**b**) Tikhonov method; (**c**) sparse-MAP method; (**d**) MRF-MAP method; (**e**) Weibull-MAP method; (**f**) the proposed hybrid-model method.

Figure 10 shows the range profile of the targets marked by the rectangular box in the results of Figure 9. Figure 10a is the profile of the real-beam echo, in which only one target can be recognized at the location of $5^{\circ }$. After super-resolution processing, two targets can be observed in all the results from Figure 10b–f. However, among these results, the method proposed in this study can achieve the highest super-resolution ratio and retain the fewest false targets. In summary, the results of the real data experiment indicate that the proposed method has a stronger ability in suppressing sea clutter and achieves an outstanding super-resolution result compared to the traditional methods.

## 5. Conclusions

This paper introduces a novel hybrid-model-based super-resolution imaging method tailored for monitoring sea surface scenes that is especially suited for ground-based or shipborne radar platforms. The approach begins with developing a new observation model that incorporates both noise and clutter. To model clutter from low-incidence angle platforms, the Weibull distribution is employed. Additionally, based on the modeling of I/Q channel noise, a likelihood function that jointly models noise and clutter is constructed under this new observation model. For complex coastal scenes, Markov distribution with two-dimensional spatial representation capabilities is utilized to model non-stationary scenes. Furthermore, square terms are introduced to bolster the algorithm’s robustness against outliers. The focus is on the processing of intricate sea surface scenes captured by ground-based or shipborne radar in contrast to existing super-resolution imaging methods for sea surface targets. Targeted and physically interpretable models are adopted for noise and clutter, effectively mitigating their impact on super-resolution imaging. Finally, simulation experiments and real data processing were used to validate that the algorithm proposed in this paper excels in scene contour recovery, noise suppression, and combating clutter interference.

## Figures and Tables

**Table 1:** Experiment parameters.

Parameters	Values	Parameters	Values
Carrier frequency	9.5 GHz	Velocity of the platform	50 m/s
Pulse repetition frequency	2000 Hz	Signal bandwidth	25 MHz
Grazing angle	$5^{\circ }$	Pulse width	40 us
Near range	8 km	Main-lobe beam width	$2^{\circ }$
Antenna scanning area	−10${}^{\circ }$∼$10^{\circ }$	Antenna scanning velocity	$60^{\circ }/s$

**Table 2:** Comparison of numerical results at SCNR = 10 dB.

Methods	ReErr	SSIM
Tikhonov	0.7132	0.6307
Sparse-MAP	0.8831	0.5260
MRF-MAP	0.7093	0.6608
Weibull-MAP	0.5113	0.7906
The proposed method	0.2761	0.9214

## Data Availability

Data are contained within the article.
